# Fast Fabrication of Nanostructured Films Using Nanocolloid Lithography and UV Soft Mold Roller Embossing: Effects of Processing Parameters

**DOI:** 10.3390/polym13030405

**Published:** 2021-01-27

**Authors:** Demei Lee, Ya-Ling Tang, Shih-Jung Liu

**Affiliations:** 1 Department of Mechanical Engineering, Chang Gung University, Taoyuan 33302, Taiwan; dmlee@mail.cgu.edu.tw (D.L.); vellick27candy@yahoo.com.tw (Y.-L.T.); 2 Department of Orthopedic Surgery, Bone and Joint Research Center, Chang Gung Memorial Hospital-Linkou, Taoyuan 33305, Taiwan

**Keywords:** nanocolloid lithography, UV soft mold roller embossing, nanofeatured film, replication

## Abstract

We report the fabrication of nanofeatured polymeric films using nanosphere lithography and ultraviolet (UV) soft-mold roller embossing and show an illuminative example of their application to solar cells. To prepare the nanofeatured template, polystyrene nanocolloids of two distinct sizes (900 and 300 nm) were overlaid on silicon substrates using a spin coater. A lab-made soft-mold roller embossing device equipped with a UV light source was adopted. A casting method was employed to replicate the nanofeatured template onto polydimethylsiloxane, which was used as the soft mold. During the embossing procedure, the roller was driven by a step motor and compressed the UV-curable resin against the glass substrate to form the nanofeatured layer, which was subsequently cured by UV radiation. Polymer films with nanoscaled features were thus obtained. The influence of distinct processing variables on the reproducibility of the nanofeatured films was explored. The empirical outcomes demonstrate that UV soft-mold roller embossing offers a simple yet potent way of producing nanofeatured films.

## 1. Introduction

Nanotechnology confines the understanding of the basic physics, chemistry, biology, and technology of nanoscaled objects, and has considerably improved various technical and industrial sectors including information, medicine, and energy, etc. [1]. Amidst these, polymeric films with nanofeatured surfaces have found various applications such as antimicrobial, scratch-resistant, electrically conductive, information processing, optoelectronics, and integrated optics [2].

To fabricate nanofeatured films, several fabrication methods were proposed, including laser ablation, photolithography, micro-jet manufacture, and hot embossing, etc. [3]. Amidst these technologies, hot embossing is the highly employed method for reproducing a microfeatured array [4]. The process offers the advantages of molding/patterning large areas with nanofeatures, and refrains the limitations associated with lithography that requires time-consuming exposure and development [5]. Despite the advantages associated with hot embossing, the plastic film for embossing needs to be heated to above the glass transition temperature. The core of the film is over-softened, which leads to a long cycle time for the process. Meanwhile, the batch-wise process of hot embossing also makes it an unsuitable scheme for mass reproduction. Roller embossing, a continuous procedure that offers large area fabrication with low cost and high throughput [6,7], was proposed with the aim of reducing the processing time. Additionally, to further minimize heating and cooling times, an ultraviolet (UV) embossing method [8,9] was used. This technique employs a UV-curable photopolymer and utilizes UV radiation to cure the photopolymer. This low pressure and temperature UV embossing scheme allows significant reduction of the cycle time and accelerates fabrication speeds.

**Figure 1:**
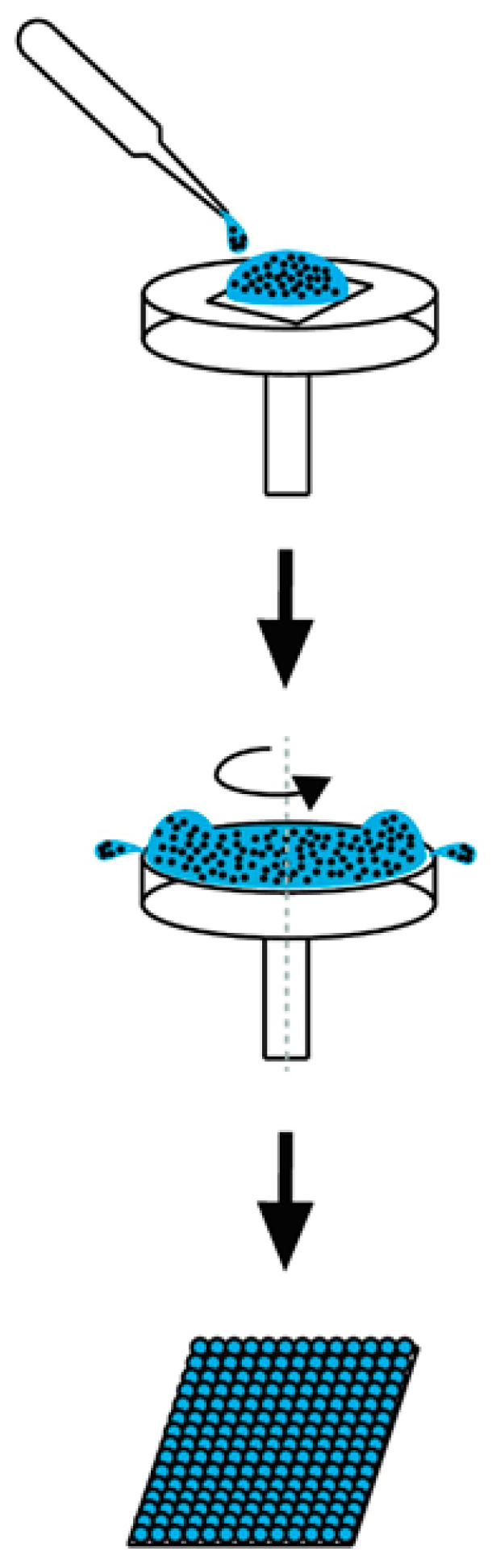
The schematic spin coating process.

Nanocolloid lithography [10,11] is a simple and inexpensive nanofabrication technique that is competent to produce various nanocolloidal structures and well-ordered two-dimensional nanofeatured arrays, which can act as a template for the embossing process. As shown schematically in Figure 1, a flat substrate was overlaid with a suspension incorporating monodisperse nanocolloids after being chemically treated so as to promote its hydrophilicity. After drying, a nanofeatured layer was then formed.

In this study, we report the fabrication of nanofeatured films using nanocolloid lithography and UV soft mold roller embossing, and show an illustrative example of their application to solar cells. To prepare the nanofeatured template, polystyrene nanospheres (900 and 300 nm) were overlaid on a silicon substrate employing a spin coater. A soft-mold roller embossing device equipped with a UV exposure source previously built in our lab was adopted. A casting method was also employed to replicate the nanofeatured template onto polydimethylsiloxane, which was used as the soft mold. During the embossing procedure, the roller was driven by a step motor and compressed the UV-curable resin against the glass substrate to form the nanofeatured layer, which was subsequently cured by UV radiation. Polymer films with nanofeatured surfaces were obtained. The replicability and the surface roughness of embossed films were characterized. Additionally, the influence of distinct processing variables on duplicated film quality was evaluated.

## 2. Materials and Methods

### 2.1. Materials

Commercially available polystyrene colloidal nanospheres with two distinct sizes (900 and 300 nm) were acquired from micro-Particles GmbH. Ethanol was purchased from Sigma-Aldrich, St. Louis, MO, USA. The polydimethylsiloxane (PDMS) pre-polymer solution (Model SYLGARD 184) and cross-linker were purchased from Dow Corning, Midland, MI, USA. Dispersant (Model DS-UPS-Aw series) was obtained from Golden Innovation Business Co, Taipei, Taiwan, while ultraviolet curable epoxy resin (Model FL171-10) was acquired from Everwide Chemical Co, Taipei, Taiwan. In addition, hexane was purchased from JT-Baker, Phillipsburg, NJ, USA.

### 2.2. Preparation of Nanofeatured Template Using Nanosphere Lithography

To self-assemble the colloidal array, 300 nm nanosphere latex was overlaid on a silicon substrate (6.5 cm by 11.5 cm) employing a spin coater (SP-M3-P, Apisc Co., Taipei, Taiwan). Some test trials were completed so as to identify the optimum processing conditions. As shown in Table 1, the influence of processing variables on self-assembly of 300 nm colloid arrays, including percentage of dispersant, spin time, and spin speed, on assembled array properties were investigated. The percentage of dispersant was either 7, 10, 30, or 40 wt%. To spin coat the array, the spin speeds were set at either 2000, 3000, 4000, or 5000 rpm, while the spin times were either 20, 30, 40, or 50 s. A surfactant solution with distilled (DI) water to ethanol in a ratio of 1:1 (*v*/*v*) was used, while the ratio of surfactant solution to 300 nm nanosphere in a ratio of 1:2 (*w*/*w*) was adopted.

The spin coating of the 900 nm colloid array followed the same scheme for that of the 300 nm, except that a multi-stage spin coating was utilized, for example, the substrate was subject to a spin speed of 500 rpm for 30 s followed by a spin speed of 1500 rpm for 30 s and a spin speed of 2000 rpm for 60 s. Table 2 lists the processing parameters employed in the tests.

### 2.3. Preparation of the Soft Mold

The soft mold was obtained by pouring the polydimethylsiloxane (PDMS) pre-polymer solution (Dow Corning SYLGARD 184) onto the nanosphere arrays, which acted as a template. The ratio of PDMS pre-polymer solution to cross-linker was 10:1 (*w*/*w*), while the ratio of PDMS to hexane was either 2:1, 1:1, 1:2, or 1:5 (*w*/*w*). After curing, the PDMS mold was exfoliated from the template. The mold with a nanocavity array was placed into acetonitrile and subject to sonication for 30 min to remove the residual PS spheres stuck on the mold. A soft mold (a dimension of 1 × 1 cm^2^ and a thickness of 0.5 cm) with nanocavities was thus obtained.

### 2.4. Roller Embossing of Nanofeatured Films

**Figure 2:**
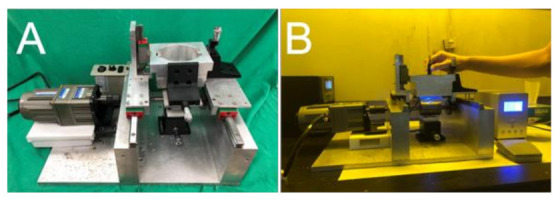
Photos of an (**A**) ultraviolet (UV) soft mold roller embossing device, (**B**) UV soft mold embossing process.

Roller embossing experiments were conducted on a lab-made UV roller embossing device [3]. The device consists of a UV light source, a soft mold roller stamp that has a diameter of 50 mm, a movable table actuated via a speed-adjustable step motor, and a container that accommodates the UV-curable polymeric solutions (Figure 2). The utmost power of the UV light (BWL-100, ByWell Materials Co, New Taipei City, Taiwan) is 3900 mW/cm^2^ with a wavelength of 365 nm. Two levels of UV radiation, 530 and 3900 mW/cm^2^, were employed, while three shifting speeds of the table, 5.2, 13.1, and 20.9 mm/s, were adopted.

The embossing pressures were altered by varying the distance between the roller stamp and glass substrate. Two Z-stages situated over the movable roller were used to adjust the distance and embossing pressure. Three distances (−200, 0, and 100 μm) were adopted for the roller embossing procedure. The distance of 0 mm denotes a mild contact while a negative distance indicates interference between the roller stamp and glass substrate. Furthermore, the distance between the roller stamp and the liquid photopolymer container below the roller could also be altered via the Z-stage.

In the embossing procedure, the UV curable epoxy resin with a refractive index 1.45 at a wavelength of 365 nm and a viscosity of 320 to 470 cps at 25 °C was first kept in the container. As the roller stamp rotated, the stamp (soft mold) came into contact with the UV-curable resin, and the liquid photopolymer was squeezed into the nanocavities of the mold on the roller. Once the roller was in contact with the glass substrate, a polymer film with nanofeatures was then formed on the substrate post-UV radiation. After being peeled off of the glass substrate, nanofeatured films were obtained.

## 3. Results and Discussion

### 3.1. Fabrications of Nanocolloidal Template

Table 1 and Table 2 list the processing variables and values used in the experiments. By changing one variable each time while keeping the other parameters intact, we were able to examine the influence of various processing parameters on the spin coating of 300 and 900 nm nanosphere arrays.

**Figure 3:**
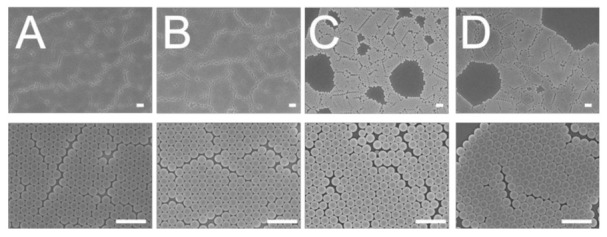
Influence of dispersant percentage on the assembly of 300 nm nanosphere arrays, (**A**) 7%, (**B**) 10%, (**C**) 30%, (**D**) 40%. (Spin speed was mainted 3000 rpm and spin time was 30 s. Scale bar: 1 μm; Magnification: top—6000×, bottom—25,000×).

The influence of weight percentage of dispersant on the centrifuged 300 nm colloidal array is shown in Figure 3. Similar to all naturally occurring crystals, the nanocolloid arrays displayed distinct defects that emerge owing to nanosphere polydispersity, site randomness, vacancies, line defects, and polycrystalline domains [12]. While the 7% dispersant assembled array showed an overlapping nanosphere layout and the 30% and 40% dispersant assays exhibited vacancies, the 10% dispersant array demonstrated a more uniform distribution of the nanospheres. Attractive capillary forces, convective transport of the nanospheres, concentration of the colloid suspension, and centrifugal force all play an important role in determining the ordering and quality of the resulting arrays. An appropriate percentage of dispersant thus helped uniformly distribute the nanoparticles onto the silicon substrates.

**Figure 4:**
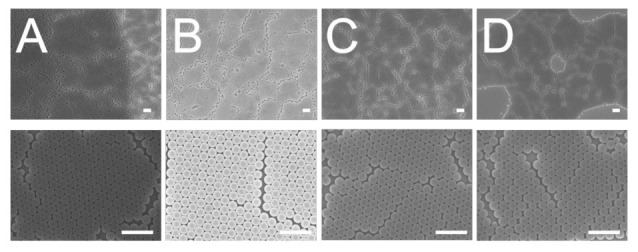
Influence of spin speed on the assembly of 300 nm nanosphere arrays, (**A**) 2000 rpm, (**B**) 3000 rpm, (**C**) 4000 rpm, (**D**) 5000 rpm (disperant percentage was 10% and spin time was 30 s. Scale bar: 1 μm; Magnification: top—6000×, bottom—25,000×).

**Figure 5:**
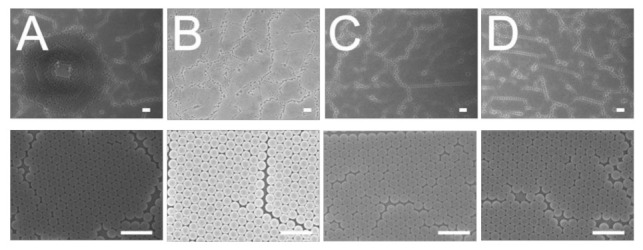
Influence of spin time on the assembly of 300 nm nanosphere arrays, (**A**) 20 s, (**B**) 30 s, (**C**) 40 s, (**D**) 50 s (disperant percentage was 10% and spin speed was 3000 rpm. Scale bar: 1 μm; Magnification: top—6000×, bottom—25,000×).

Figure 4 displays the distributions of the 300 nm nanospheres on the substrates that were subject to different centrifugation speeds. Obviously, compared to the other arrays, the array spun with 3000 rpm in Figure 4b shows the most uniform nanosphere distribution. Meanwhile, Figure 5 shows the characteristics of coated arrays subjected to various spin times. The array spin coated with 30 s centrifugation time in Figure 5B exhibited the most uniform nanosphere distribution characteristic. During spin coating, the centrifugal force was critical in affecting the quality of self-assembled arrays. Adopting a too-low spin speed or spin time could not spin out the nanospheres, and in this situation, particle clusters might be observed. However, if the centrifugation speed or time was too high, the centrifugal forces might have overspun the particles, which would have led to vacant defects.

**Figure 6:**
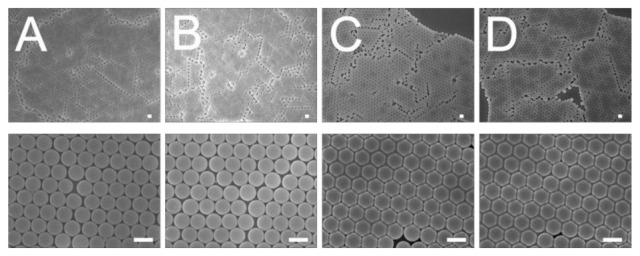
Influence of dispersant content on the assembly of 900 nm nanosphere arrays, (**A**) 7%, (**B**) 10%, (**C**) 30%, (**D**) 40%. (Spin conditions were 500 rpm for 30 s followed by 1500 rpm for 30 s, and 2000 rpm for 60 s. Scale bar: 1 μm; Magnification: top—3000×, bottom—15,000×).

The influence of weight percentage of dispersant on the centrifuged 900 nm colloidal array is shown in Figure 6. It was found that the 7% dispersant caused the nanospheres to assemble with the least slip dislocations (top image of Figure 6A). Compared to the other solutions, the 7% dispersant solution had the lowest viscosity that facilitated the convective transport of the nanospheres; therefore, it was easier for the nanospheres to be assembled in an ordered manner during the spin coating process.

**Figure 7:**
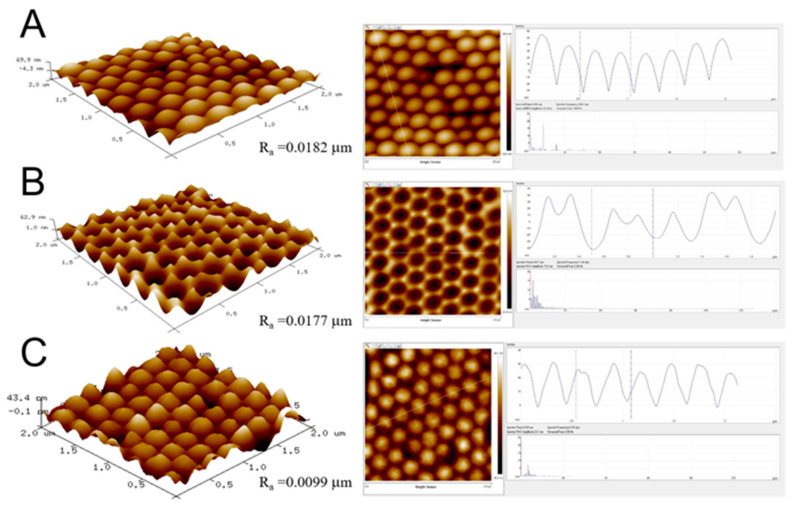
Atomic force microscopy (AFM) surface profiles of (**A**) an assembled 300 nm nanosphere array, (**B**) a replicated soft mold, and (**C**) an embossed nanofeatured film.

**Figure 8:**
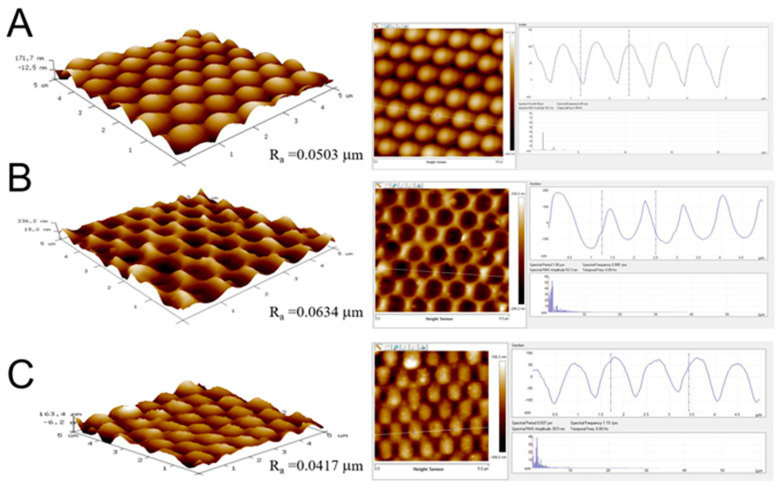
AFM surface profiles of (**A**) an assembled 900 nm nanosphere array, (**B**) a replicated soft mold, and (**C**) anembossed nanofeatured film.

With a combination of proper processing conditions, namely a dispersant content of 7%, a spin speed of 3000 rpm, and a spin time of 30 s for the 300 nm nanocolloids, and a dispersant content of 7% and spin conditions of 500 rpm for 30 s followed by 1500 rpm for 30 s, and 2000 rpm for 60 s for the 900 nm nanocolloids, nanosphere arrays of 300 and 900 nm with good dimensional uniformity could be obtained. The arrays were characterized by an atomic force microscope (AFM), and Figure 7A and Figure 8A show the measured image of the assembled 300 and 900 nm nanosphere arrays, respectively. The average surface roughness (Ra) for the 300 nm array was 18.2 nm, while the roughness for the 900 nm array was 50.3 nm. The empirical outcomes demonstrate that the spin coating technique could successfully cause self-assembly of the nanospheres onto the silicon substrate via uniform distribution.

### 3.2. Preparation of Soft Molds

**Figure 9:**
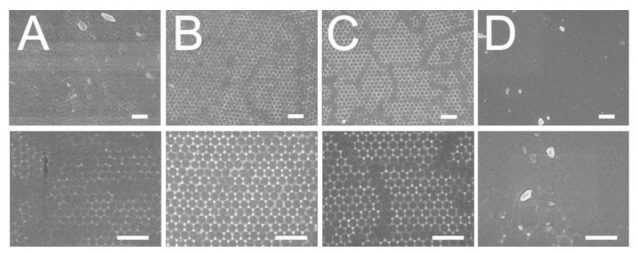
Influence of polydimethylsiloxane (PDMS) to hexane ratio, (**A**) 2:1, (**B**) 1:1, (**C**) 1:2, (**D**) 1:5, on the replication of 300 nm nanosphere array onto the soft mold. (Scale bar: 1 μm; Magnification: top—12,000×, bottom—25,000×).

**Figure 10:**
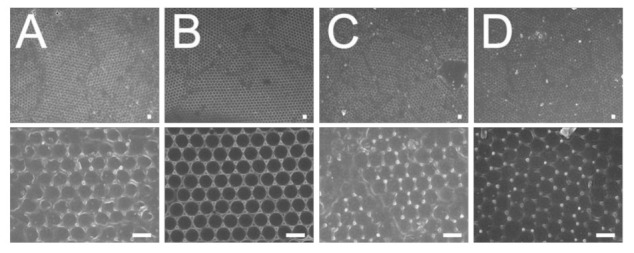
Influence of PDMS to hexane ratio, (**A**) 2:1, (**B**) 1:1, (**C**) 1:2, (**D**) 1:5, on the replication of 900 nm nanosphere array onto the soft mold. (Scale bar: 1 μm; Magnification: top—3000×, bottom—15,000×).

The influence of PDMS to hexane ratio on nanocavity replication on the soft mold was examined. Figure 9 and Figure 10 show the scanning electron microscopy (SEM) images of prepared soft molds with 300 and 900 nm nanocavities, respectively. The SEM image in Figure 9B suggests that the use of a 1:1 ratio of PDMS to hexane resulted in the most uniform 300 nm nanocavity distribution of the PDMS soft mold. Meanwhile, the SEM image in Figure 10B also shows that the 1:1 PDMS to hexane ratio produced a soft mold with the most uniform 900 nm nanocavities. During the PDMS casting process, PDMS/hexane solution flowed into the surface of nanosphere array to replicate the nanofeatures. After the hexane evaporated, the PDMS mold was obtained. With a percentage of hexane that was too high, the nanofeature would have deteriorated due to the evaporation of hexane. However, if the hexane percentage was too low, the viscosity of the solution would have increased accordingly. It then becomes more difficult for the PDMS/hexane solution to conform to nanosphere array and replicate the structures.

The average surface roughnesses (Ra) for 300 and 900 nm soft molds (in Figure 9B and Figure 10B) were 17.7 and 63.4 nm, respectively. The AFM analysis results suggest that the nanofeatured arrays could be successfully replicated on the PDMS soft molds with good distribution.

### 3.3. Roller Embossing of Nanofeatured Films

**Figure 11:**
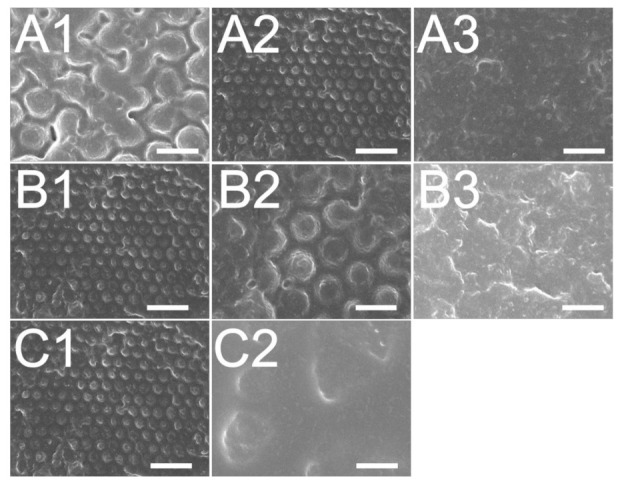
Effect of (**A**) roller stamp/glass substrate distance; (**A1**): +100 μm, (**A2**): 0 μm, a3: −200 μm, (**B**) rolling speed; (**B1**): 5.23 mm/s, (**B2**): 13.08 mm/s, (**B3**): 20.93 mm/s, (**C**) UV dose; (**C1**): 3900 mW/cm^2^, (**C2**): 530 mW/cm^2^, on the fabrication of 300 nm nanofeatured films.

The influence of distinct processing variables, including table moving speed, distance between the roller stamp and the glass substrate, and UV dose, on the formation of the surface nanostructures were evaluated. Table 3 lists these processing variables and the variable values used in the test trials. By altering one of the variables in each test trial while maintaining the other ones at steady values, we were able to estimate the influence of each variable on the replicability of embossed films. Figure 10 and Figure 11 show the SEM images of embossed 300 and 900 nm featured films, respectively, that were subject to various processing conditions.

**Figure 12:**
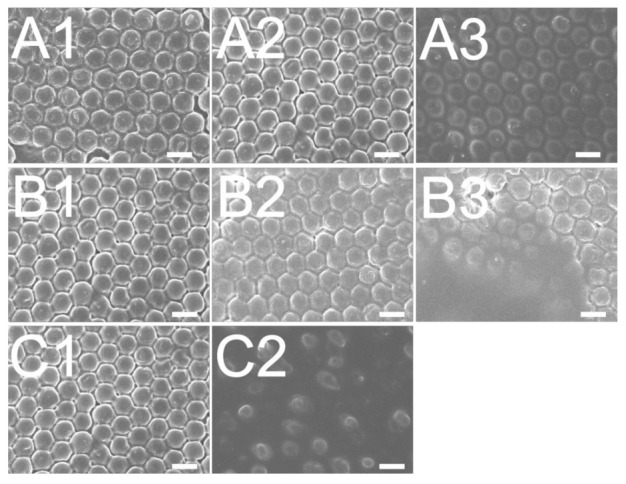
Effect of (**A**) roller stamp/glass substrate distance; (**A1**): +100 μm, (**A2**): 0 μm, (**A3**): −200 μm, (**B**) rolling speed; **B1**: 5.23 mm/s, (**B2**): 13.08 mm/s, (**B3**): 20.93 mm/s, (**C**) UV dose; (**C1**): 3900 mW/cm^2^, (**C2**): 530 mW/cm^2^, on the fabrication of 900 nm nanofeatured films.

The influence of the roller stamp/glass substrate distance was examined and shown in Figure 11A and Figure 12A. The experimental result in Figure 11(A2) and Figure 12(A2) suggest that the distance of 0 μm embossed films yielded the most superior replicability. The conformability of the film to the mold geometry is the primary consideration for the roller embossing of polymeric products. In the embossing procedure, a pressure was imposed onto the roller stamp to drive the liquid photopolymer to flow and fill the nanocavities. The imposed pressure was raised by decreasing the distance between the roller stamp and the glass substrate. As the + 100 μm distance was used, the pressure was not high enough to replicate the nanofeatures. However, if the pressure had been too high (when the –200 μm distance was used), the soft mold could have been disfigured, pressing excessive photopolymer into the nanocavity. As the pressure was removed after the glass substrate was driven forward by the step motor, the rubbery soft mold restored its geometry and compressed the superabundant liquid photopolymer outwards. Consequently, a distance of 0 μm embossed polymeric films with the most superior quality was obtained.

Figure 11B and Figure 12B show the influence of the table’s moving speed on the reproducibility of 300 and 900 nm nanofeatures, respectively. The results suggest that the embossed quality of the nanofeature decreased with the moving speed. This finding might be due to the fact that in roller embossing, a pressure is imposed onto the glass substrate adjacent to the soft mold for some time so as to compress the liquid photopolymer into the nanocavity for nanostructure conformity. If the table is moving too fast, the photopolymer may not possess enough time to entirely fill the cavity. Roller embossing with the lowest moving speed thus molded polymer films with the best product quality.

The influence of various UV doses on film quality was also evaluated. The experimental result in Figure 11C and Figure 12C suggest that the reproducibility of embossed surface nanostructure improves with the UV light dose. In roller embossing, once the nanofeature array was created on the substrate, the features require UV radiation to cure and solidify the liquid photopolymer. Nevertheless, if the selected UV dose is not high enough, the liquid photopolymers may not absorb sufficient energy to photo-cure the materials. The liquid photopolymer subsides and the replicated quality lessens accordingly.

By adopting the optimum conditions, 300 and 900 nm nanofeatured films were successfully embossed. The average surface roughnesses (Ra) for 300 and 900 nm nanofeatured films (in Figure 7C and Figure 8C, respectively) measured by the AFM were 9.9 nm and 41.7 nm, respectively. The AFM results suggest that the nanofeatured arrays could be successfully embossed on the polymeric films with good distribution.

### 3.4. Characterization of Embossed Films

**Figure 13:**
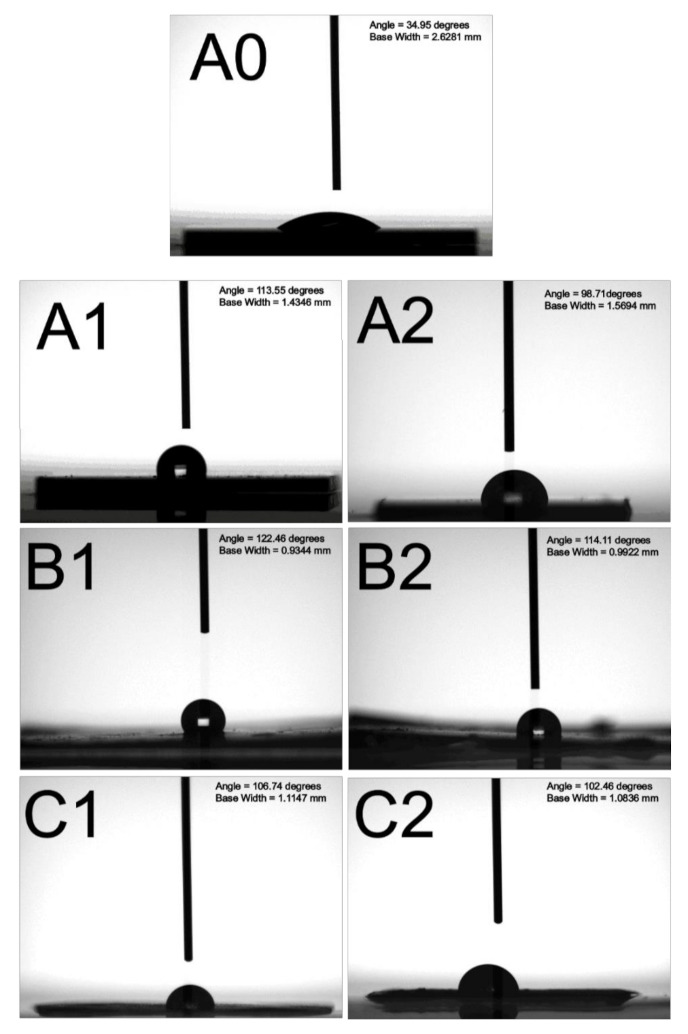
Water contact angles of (**A0**) silicon substrate—34.95°, (**A1**) assembled 300 nm nanosphere array—113.55°, (**A2**) assembled 900 nm nanosphere array—78.56°, (**B1**) replicated 300 nm soft mold—122.46°, (**B2**) replicated 900 nm soft mold—114.11°, (**C1**) embossed 300 nm featured film—106.74°, (**C2**) embossed 900 nm featured film—102.46°.

The water contact angles of assembled nanosphere arrays, replicated PDMS soft molds, and embossed nanofeatured polymeric films were evaluated. Figure 13 displays the measured water contact angles. The water contact angle for the silicon substrate was 34.95°, and the contact angles for the assembled 300 and 900 nm arrays were 113.55° and 98.71°, respectively. Despite the fact that the silicon substrates showed hydrophilic behaviors, the assembled nanostructured arrays displayed an additional hydrophobic pattern. Additionally, the water contact angles for the replicated soft molds of 300 and 900 nm nanofeatures were 122.46° and 114.11°, respectively, while the contact angles for the embossed nanostructured films were 106.74° and 102.46°, respectively. Clearly, the hydrophobicity of embossed films was enhanced with the nanofeatures.

**Figure 14:**
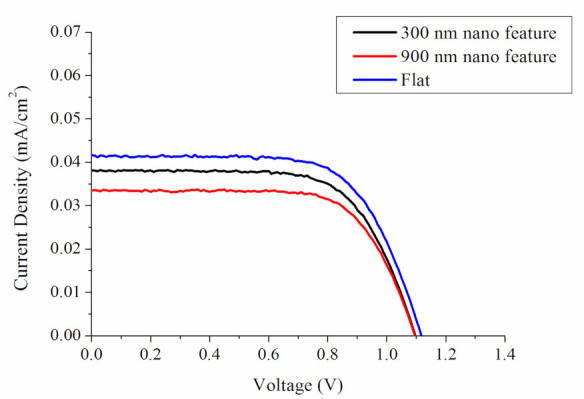
Current–voltage profiles of the solar cell with and without nanofeatured films.

To assess the anti-reflective feasibility of the nanostructured polymeric, photocurrent–voltage quantifications were completed on a lab-made solar cell and a solar simulator (Solar IV 150, Titan Electro-Optics Co., Taipei, Taiwan). Figure 14 shows the estimated current–voltage patterns of the solar cells with flat and nanofeatured films, while Table 4 lists the open circuit voltage (Voc), fill factor (FF), short circuit current (Isc), and energy conversion efficiency (Eff) for the solar cells. Clearly, all polymeric films with nanofeatures exhibit greater energy conversion efficiency than the flat films. Furthermore, the 300 nm featured films show higher conversion efficiency than the 900 nm films. The experimental outcomes verify that the nanofeatured films could potently promote the capability of solar cells.

## 4. Conclusions

This paper adopted nanosphere lithography and UV roller embossing technology for the manufacturing of polymer films with nanofeatures. The influence of various spin coating parameters on the assembly of nanocolloid arrays was explored. Furthermore, the effects of distinct processing variables on the replicability of nanofeatures onto the polymeric films were also evaluated. By adopting the appropriate processing conditions, polymer films with arrays of 900 and 300 nm nanostructures were satisfactorily manufactured. The hydrophobicity of embossed films was enhanced with the nanofeatures. In addition, all polymeric films with nanofeatures exhibit greater energy conversion efficiency of solar cells than the flat films. The 300 nm featured films also show higher conversion efficiency than the 900 nm films. The empirical outcomes in this work demonstrate that UV soft mold roller embossing offers an effective way of producing nanofeatured films employing low pressure at room temperature. This method will offer considerable advantages with regard to a shorter processing time and enhanced part quality.

## Figures and Tables

**Table 1:** The processing parameters used for self-assembly of 300 nm nanospheres.

DI Water:Ethanol (*v*/*v*)	Surfactant:300 nm PS Sphere (*v*/*v*)	Dispersant (%)	Spin Speed (rpm)	Spin Yime (s)
1:1	1:2	7	2000	20
		10	3000	30
		30	4000	40
		40	5000	50

**Table 2:** The processing parameters used for self-assembly of 900 nm nanospheres.

DI Water:Ethanol (*v*/*v*)	Surfactant:900 nm PS Sphere (*v*/*v*)	Dispersant (%)	Spin Speed (Spin Time) rpm (s)
1:1	1:2	7	500 (30) 1500 (30) 2000 (60)
		10	
		30	
		40	

**Table 3:** The processing parameters used for roller embossing of nanofeatured films.

Rolling Speed (mm/s)	Roller Stamp/Glass Substrate Distance (μm)	UV Dose (mW/cm^2^)
5.2	+100	3900
13.1	0	530
20.9	−200	

**Table 4:** Current-voltage characteristics of the solar cells subjected to polymer films with and without nanofeatures.

Film Type	V_max_ (V)	I_max_(mA/cm^2^)	V_OC_ (V)	I_SC_ (mA/cm^2^)	FF (%)	Eff (%)
Flat	0.69	1.12	0.71	1.32	0.57	5.38
300 nm nanofeature	0.67	1.09	0.70	1.44	0.62	6.02
900 nm nanofeature	0.56	1.02	0.67	1.42	0.59	5.96
